# Weed Risk Assessment for Aquatic Plants: Modification of a New Zealand System for the United States

**DOI:** 10.1371/journal.pone.0040031

**Published:** 2012-07-13

**Authors:** Doria R. Gordon, Crysta A. Gantz, Christopher L. Jerde, W. Lindsay Chadderton, Reuben P. Keller, Paul D. Champion

**Affiliations:** 1 The Nature Conservancy and Department of Biology, University of Florida, Gainesville, Florida, United States of America; 2 University of Notre Dame, Notre Dame, Indiana, United States of America; 3 The Nature Conservancy, c/o Environmental Change Initiative, University of Notre Dame, Notre Dame, Indiana, United States of America; 4 Department of Environmental Science, Loyola University Chicago, Chicago, Illinois, United States of America; 5 Freshwater Biosecurity National Institute of Water & Atmospheric Research, Hamilton, New Zealand; University of Canterbury, New Zealand

## Abstract

We tested the accuracy of an invasive aquatic plant risk assessment system in the United States that we modified from a system originally developed by New Zealand’s Biosecurity Program. The US system is comprised of 38 questions that address biological, historical, and environmental tolerance traits. Values associated with each response are summed to produce a total score for each species that indicates its risk of invasion. To calibrate and test this risk assessment, we identified 39 aquatic plant species that are major invaders in the continental US, 31 species that have naturalized but have no documented impacts (minor invaders), and 60 that have been introduced but have not established. These species represent 55 families and span all aquatic plant growth forms. We found sufficient information to assess all but three of these species. When the results are compared to the known invasiveness of the species, major invaders are distinguished from minor and non-invaders with 91% accuracy. Using this approach, the US aquatic weed risk assessment correctly identifies major invaders 85%, and non-invaders 98%, of the time. Model validation using an additional 10 non-invaders and 10 invaders resulted in 100% accuracy for the former, and 80% accuracy for the latter group. Accuracy was further improved to an average of 91% for all groups when the 17% of species with scores of 31–39 required further evaluation prior to risk classification. The high accuracy with which we can distinguish non-invaders from harmful invaders suggests that this tool provides a feasible, pro-active system for pre-import screening of aquatic plants in the US, and may have additional utility for prioritizing management efforts of established species.

## Introduction

Documented impacts of invasive non-native freshwater aquatic plant species include alterations to water chemistry, hydrologic regimes, temperature and sedimentation rates, and loss of native biodiversity [1]. Additionally, invaders can be expensive for both private and public sectors through the costs incurred by treatment of infestations, increased disease transmission, and lost opportunities for navigation, fisheries, and hydroelectric generation [1]–[4]. As an example, the invasion of water hyacinth (*Eichhornia crassipes*) into Africa’s Lake Victoria hampered navigation by growing in thick mats on the surface, which also provided extensive breeding grounds for mosquitoes, resulting in increased transmission of vector-borne diseases [5]. Although full costs are difficult to quantify, invasive aquatic plant species are responsible for an estimated $110 million in annual control costs and damage to navigation, recreation, and agriculture in the US [6]. This number may be a substantial underestimate; the state of Florida (US) alone spends roughly $20 million annually to control just one species, *Hydrilla verticillata* [7].

Although the proportion of introduced aquatic plants that cause harmful impacts is small [8], both the number of species introduced and the frequency of introductions have increased rapidly as global markets have broadened [9], [10] and as interest in water gardening has grown [11], [12]. In the US, for example, the number of households with water gardens quadrupled between 1998 and 2003, reaching an estimated value of US$1.56 billion [13], and the global trade in species for aquaria and water gardens is growing by 14% per year [14]. Although some aquatic plant species deemed to be high risk are regulated in some regions, most remain available from stores, and increasingly from on-line commercial and hobbyist sources [11].

The increased availability of aquatic plants merits particular attention because freshwater aquatic and semi-aquatic plants have a higher probability of becoming invasive than do species from terrestrial plant families [15]. This higher risk means that in many regions most aquatic invaders are derived from intentional imports. For example, 75% of the aquatic invasive plants in New Zealand were imported for horticultural use [3], as were 76% of all aquatic plants naturalized in the southern New England region of the US [16], and 85% of aquatic plants naturalized in Australia [17]. Risks of new invasions continue to increase with increasing trade [12].

The role of intentional imports in producing damaging invasions has motivated efforts to develop risk assessment tools that would allow regulation of potentially harmful species prior to their introduction [3], [18]–[21]. These tools are developed through identification of consistent patterns in traits of species that have previously become harmful invaders. Historical patterns are assumed to hold into the future, so the tool can be used as a predictive risk assessment for impacts of species not yet introduced [22]. Risk assessment tools with high accuracy give regulators the option to screen species prior to their arrival (“pre-border”) and reduce costly future invasions. This approach has been demonstrated to be both environmentally and economically advantageous [23], [24].

The most widely tested risk assessment tool for plants is the Australian Weed Risk Assessment (AWRA) [18], which is used for regulatory purposes in Australia, New Zealand, Chile, and other countries. When modified to reflect local environmental conditions, the tool has high accuracy in a range of global regions [25]. However, many of the AWRA questions are specific to terrestrial plant species, and this tool is less accurate in discriminating between aquatic invaders and non-invaders [26].

Recognizing that the AWRA might not discriminate well among aquatic species, a separate risk assessment tool was developed for New Zealand (NZAqWRA) [3], [27]. The tool has subsequently been applied in Australia and Micronesia [17], [28], [29], and recommended for adoption in Europe [30]. In New Zealand, the NZAqWRA has been used both as a pre- and post-border tool, to identify potential problem species before their introduction or naturalization, and also to prioritize established species for management.

However, the NZAqWRA has not been fully validated in New Zealand or other locations. The species tested in New Zealand were primarily those with a history of prior invasion [27]. Our goals in the work reported here were to conduct such testing, refine the tool and provide guidance for more consistent implementation in the US, and to determine whether this tool can perform as well as the AWRA does for terrestrial plant species. While we call this modified tool the US Aquatic Weed Risk Assessment (hereafter USAqWRA), our hope is that it will be similarly applicable for use across other geographies with only minor modifications. An effective tool would facilitate pre-border regulation and inform post-border management decisions useful for rapid response to new infestations and resource allocation.

## Methods

The NZAqWRA is a questionnaire-style risk assessment (*sensu* [31]) that includes 36 questions about each aquatic plant to be assessed. Questions address ecology, competitive ability, dispersal modes, reproductive capacity and mode, potential for different types of impacts (e.g., to navigation, water quality), resistance to management, and history of invasion elsewhere [3]. Answers to each question are converted to a number, with high values corresponding to qualities that make the species more likely to become invasive. The final score for a species is the sum of the values for each question.

### Developing the USAqWRA

We started with the original NZAqWRA modified only by inclusion of three questions that were added to the tool when it was applied in eastern Australia [28] and Micronesia [29] (Table S1). We then modified several NZAqWRA questions so they applied more directly to US conditions. We also developed default responses for some questions, allowing their completion in the absence of data when that lack of information could be considered informative (Table S1).

Temperature tolerance (Q. 1.1) was assessed using the on-line Climate Wizard model (http://www.climatewizardcustom.org/Global_Historical) when no explicit information for a species was found in the literature. Average 3-month low winter and high summer temperatures in the native and naturalized range were compared to temperatures in the US (http://www.climatewizardcustom.org/US_Historical/). Air temperatures were used because water temperatures were not available.

Further changes to the original NZAqWRA include the addition of a question (USAqWRA Q. 1.6) on the range of pH conditions tolerated by the species [32], and removal of a competition question that can require experimental evidence to answer (NZAqWRA Q. 3.1, [28], Table S1). We also removed a question on the extent of a species’ potential range that is not yet occupied (NZAqWRA Q. 11.1). This question is included in the NZAqWRA as part of a ‘post-border’ screening, but omitted here since the USAqWRA is primarily intended for use prior to species introduction. The final USAqWRA has 38 questions in 12 categories, with a total species score that can range between 3 and 91 (Table S1).

Not all questions could be answered for all species because of data limitations. If too few questions are answered the assessment may not be reliable because the score for each species is the sum of scores from each answered question. Based on total scores and the contribution of each question, we determined that a maximum of five questions can remain unanswered for an assessment to be considered complete (Table S1).

### Evaluating Accuracy of the USAqWRA

We evaluated the USAqWRA by assessing 130 introduced aquatic plant species that have had the opportunity to become established in the United States (Table S2). Species were included only if we found evidence that they have been in the US (in the trade and/or established) for at least 30 years [26] (i.e., we ceased searching for introduction date if a date earlier than 1980 was found). We found species by searching aquatic plant lists, local floras, herbaria, encyclopedias of horticulture and water gardening (e.g., [33]–[35]), online sources (e.g., Department of Natural Resources websites), and contacting aquatic weed scientists and horticulturalists specializing in aquarium and water garden plants [26].

Aquatic plant species were categorized as attached-floating, erect emergent, free-floating, sprawling emergent, or submerged freshwater macrophytes [36]. Wetland and riparian species were not included in this analysis. The final 130 species included all non-native species identified that met the 30 year requirement. These species have good distribution across plant families and growth forms (Table S2). The numbers of temperate (USDA Hardiness Zone ≤7) [37] and tropical (Zone >7) non-native species were similar (28%–43%) in each invasion category (see below).

We developed a three tier *a priori* scale of impacts [18] for the 130 species based on their invasion history in the US: non-invader (no evidence of establishment outside of cultivation; n = 60), minor invader (established with no described ecological impacts; n = 31), and major invader (established with documented ecological impacts; n = 39). While some of the species categorized as non-invaders may be naturalized somewhere in the US, or minor invaders may in fact be increasing in abundance or impact, we used the best information in published literature and web-based searches to make the determination.

Twenty additional species (10 major invaders and 10 non-invaders) were selected to validate the USAqWRA (Table S3). Species were selected as described above, except that we included species that had been introduced to the US after 1980 within the invader category. As species in this group are already spreading into new habitats, the date of introduction clearly has not limited their expression of invasiveness.

The USAqWRA was developed to be used as a screening tool for species prior to their establishment in the US. For this reason, the only acceptable field data to test the tool are from outside the US except where data were from experiments in controlled facilities (e.g., greenhouse experiments) or on basic morphological traits (e.g., formation of floating leaves, rhizomes) that are independent of location, consistent with guidance for application of the AWRA [38]. For the validation assessment, where the goal was to evaluate accuracy of the tool on a group of species with known invasiveness, we used data on species from within the US where it was available (e.g., *Glossostigma cleistanthum, Glyceria declinata,* Table S5).

### Data Analysis

One-way Analysis of Variance (ANOVA) was used to test for differences in USAqWRA scores among the three categories of species (non-invaders, minor invaders, and major invaders). We determined the performance of the USAqWRA at different score thresholds by systematically evaluating the accuracy (percent of species correctly classified) resulting from all possible threshold score values (3 to 91) that could be used to distinguish non-invaders from invaders. We identified the threshold that maximized the classification accuracy using this analysis.

The standard way to evaluate the performance of a risk assessment tool is to report how accurately it categorizes species in the test dataset relative to their true categories of impact [31]. Although useful for identifying thresholds for classification, this approach can introduce a sampling issue because the representativeness of the test dataset to the true population of species that has been introduced cannot be determined. Because we accepted all invaders, established species, and non-invaders that met our criteria (see above), estimates based on our test dataset of the accuracy for each of these groups separately should be high. However, because we don’t know that the proportion of species in each class is the same as for the total population of species introduced (the base-rates, *sensu* [39]), using only accuracy to evaluate the performance of the risk assessment tool can be misleading. Instead, we used Receiver Operating Characteristic (ROC) curve analysis, and calculated the Area Under the Curve (AUC) [40], which is independent of the proportion of invaders and non-invaders included [41], as a metric of performance. An AUC score of 1.0 would indicate that the tool perfectly discriminates between invaders and non-invaders, while values near 0.5 indicate no discrimination capability [42]. Because we cannot predict whether minor invaders will become more invasive over time, we compared the AUC when minor invaders were classified with non-invaders with when they were classified with major invaders.

## Results

**Figure 1:**
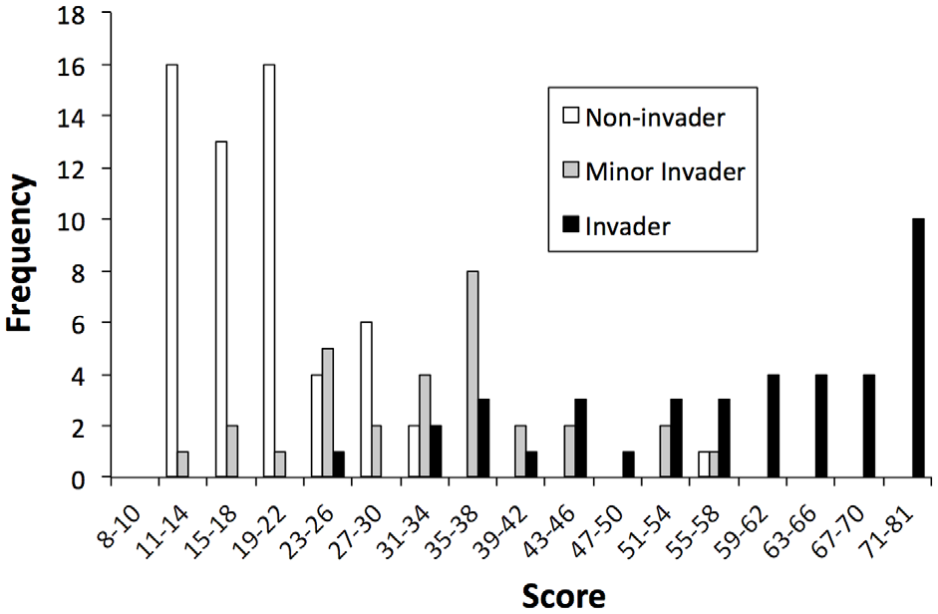
Scores for the 127 species tested using the US Aquatic Plants Weed risk assessment. See text for description of non-, minor and major invader.

Sufficient data were available to assess 127 of the 130 species (Fig. 1; Table S4). The remaining three species (*Ammannia senegalensis, Ceratophyllum submersum,* and *Hygrophila corymbosa*) could not be assessed because each had ≥5 unanswered questions; these species are not considered further. Scores for assessed species ranged from 10 to 81, with an overall mean of 34.2 and median of 28. Non-invaders (n = 58; mean (S.D.) = 19.2 (7.2)) scored significantly lower on average than minor invaders (n = 30; 32.6 (11.6)), which scored significantly lower than major invaders (n = 39; 58.6 (15.1); F = 145.15, df = 2, p<0.0001).

One non-invader (*Limnocharis flava*, managed as a potential major invader in some locations; http://www.dpi.qld.gov.au/documents/Biosecurity_EnvironmentalPests/IPA-Limnocharis-PP141.pdf), scored more than 20 points above the others in this group (Table S4). Ninety-five percent (55/58) of the non-invaders scored below 30. All species scoring 60 or higher were major invaders (Table 1; Fig. 1). Eighty-five percent (33/39) of major invaders had scores of 40 or higher, while the same proportion of minor invaders (25/30) scored below 40 (Table 1; Fig. 1).

**Table 1:** Accuracy of the USAqWRA when thresholds scores for species with low and high probability of becoming invasive are developed and intermediate scores indicate species requiring further evaluation.

	Low risk (score <31)	Evaluate further (score = 31–39)	High risk (score >39)	Total # Species
**Major Invader**	2% (1)	13% (5)	85% (33)	39
**Minor Invader**	37% (11)	47% (14)	16% (5)	30
**Non-invader**	95% (55)	3% (2)	2% (1)	58
		17% (21/127)		127
**Correctly**	82–99%		85–97%	88–93%
**predicted** 1	(55/67–66/67)		(33/39–38/39)	(93/106–99/106)

1 Correct prediction ranges, all averaging 91%, are calculated with minor invaders included as either non-invaders or major invaders without species requiring further evaluation.

**Figure 2:**
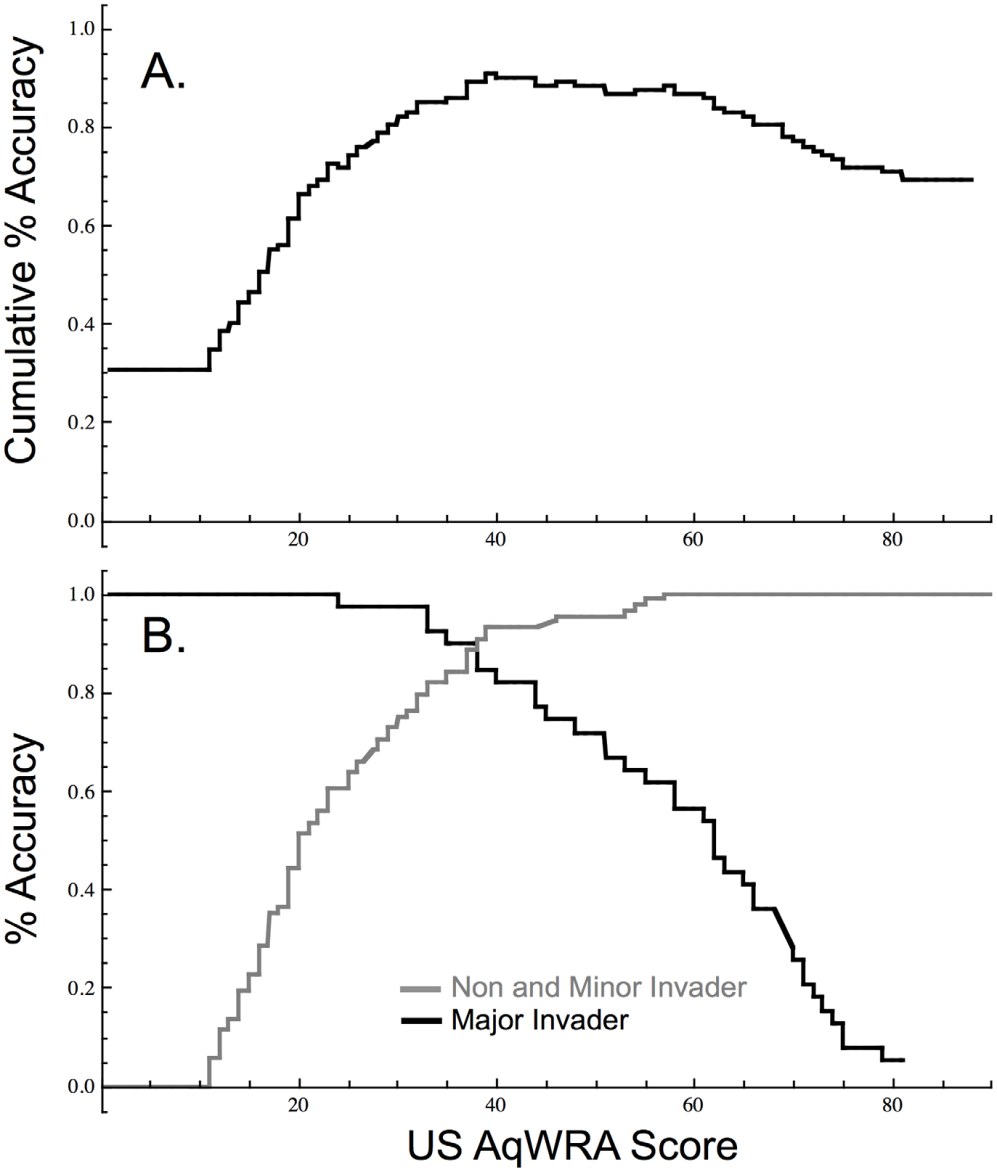
Accuracy of the USAqWRA model for the US for non-invaders and minor invaders combined, versus major invaders (n = 127). A. Cumulative percent accuracy, maximized at 90.6% at a threshold score of 40 differentiating the two groups. B. Independent percent accuracy for the non-invaders and minor invaders combined, versus major invaders.

If only non-invaders and major invaders are considered, a threshold score of 32 (scores >32 indicate high risk) differentiates each group in this dataset from the other with 97% accuracy. Given that the actual proportions of imported species in each group are unknown, as are the true categorizations of minor invaders (see above), our AUC analysis is a more conservative approach. When minor invaders were grouped with non-invaders (Fig. 2A) the USAqWRA distinguished between non-invaders and invaders with 91% overall accuracy (AUC = 0.96). Species with scores of 40 and above are predicted to be major invaders while species with scores lower than 40 are predicted to be non-invaders. Using this threshold, the USAqWRA correctly identifies major invaders 85% of the time, and non-invaders (including minor invaders) 93% of the time (Fig. 2B).

**Figure 3:**
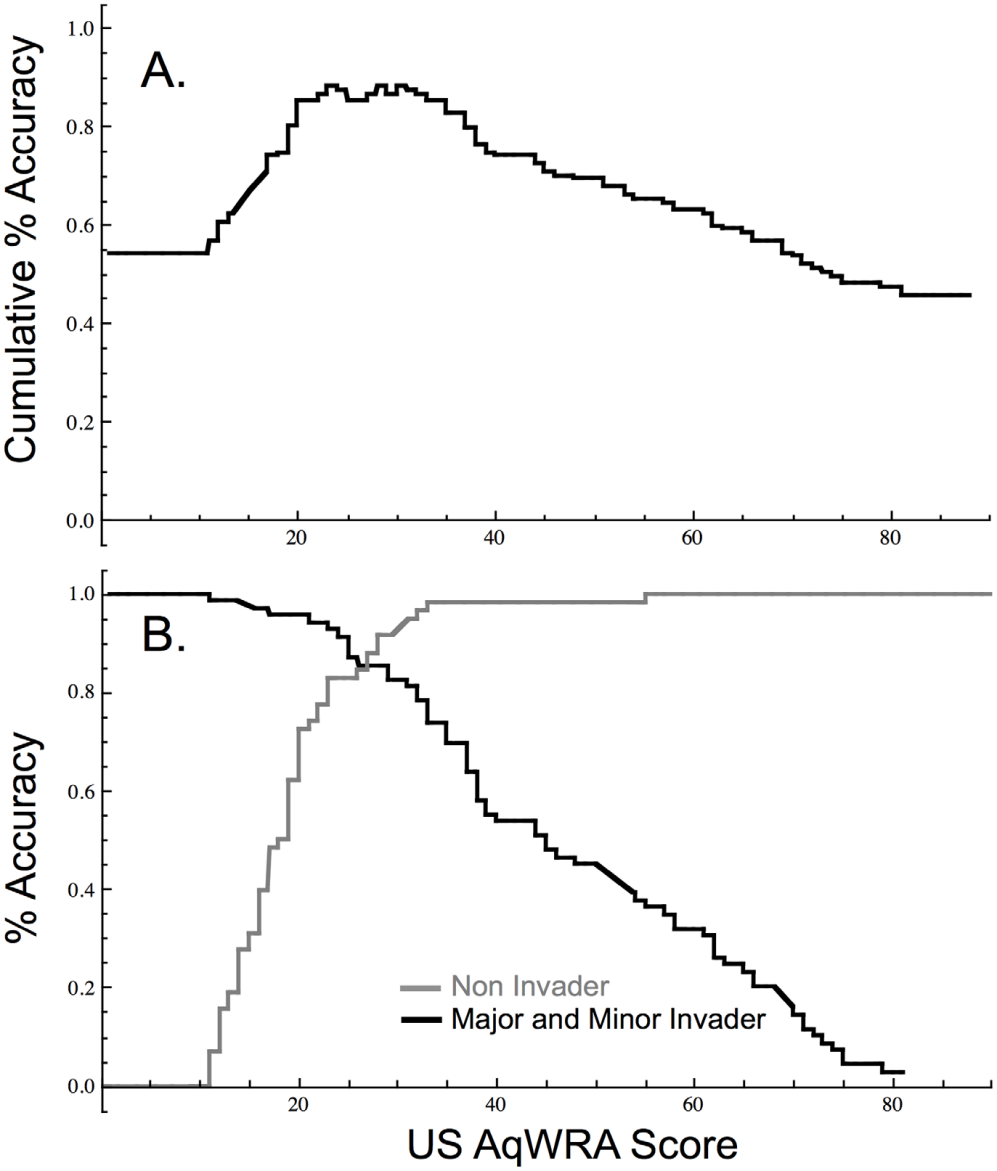
Accuracy of the USAqWRA model for the US for non-invaders versus minor and major invaders combined (n = 127). A. Cumulative percent accuracy, maximized at 88.2% at threshold scores of 24, 29, and 31 equally differentiating the two groups. B. Independent percent accuracy for the non-invaders and minor invaders combined, versus major invaders.

When minor invaders are grouped with major invaders, the overall performance is slightly lower (88%, AUC = 0.93; Fig. 3A). Under these conditions, the threshold is less well defined; scores of 24, 29, and 31 all distinguish between invaders (major and minor) and non-invaders with equivalent overall accuracy (Fig. 3B). When the threshold is 24 (species with scores ≥24 predicted to be invasive), the accuracy for major and minor invaders is 93% (100% of the major invaders), while that for non-invaders is 83%. When the threshold is 31, those values are 83% and 95%, respectively (Fig. 3B), although 97% of major invaders are correctly classified. Regardless of how the minor invaders are grouped, all AUC values are significantly different from 0.5 (p<0.001), indicating that the USAqWRA distinguishes among species in different invasiveness categories.

The score range with greatest overlap between minor and major invaders (Fig. 1) and AUC thresholds that maximize differentiation of these groups depending on classification of the minor invaders is 31–39. If this range were used to identify species requiring further evaluation, the average overall accuracy for low and high risk outcomes is 91% (Table 1). Under these conditions, 21 (17%) of the species would require further evaluation (Tables 1, S4).

The ten invasive validation species scored from 35–74, and the ten non-invader validation species scored from 15–31 (Table S5). If a threshold of 40 is used, 100% of the non-invaders, and 80% of the invaders are correctly classified. Using any of the thresholds when major and minor invaders were grouped as invaders, 100% of the validation invaders are correctly identified, while 70% (threshold of 24) or 90% (thresholds of 29 or 31) of non-invaders are correctly identified. If scores of 31–39 require further evaluation, three of the validation species would fall in this category with the remainder predicted to have high or low risk with 100% accuracy (Table S5).

## Discussion

The USAqWRA distinguished between non-invaders and major invaders among aquatic plant species with higher accuracy than the AWRA does for terrestrial [25] or aquatic [26] species. This result is strengthened because the accuracy assessment includes minor invaders, which have traditionally been excluded from accuracy calculations because of uncertainty in their long-term impacts (see [25] and references therein). Additionally, the tool is feasible: sufficient data were available to evaluate all but 2% of the species included.

Overall accuracy of the USAqWRA was roughly 90% for this dataset regardless of whether minor invaders were included with the non-invaders or major invaders (Table 1). The most precautionary approach to invader identification, while maintaining maximum overall accuracy (Fig. 3), would be to use a threshold score of 24 (i.e., scores ≥24 indicate high invasiveness risk), correctly identifying 93% of the invaders as high risk (100% of major; 86% of minor), and 83% of the non-invaders as low risk. An alternative and less precautionary approach classifies minor invaders with non-invaders, resulting in an accuracy-maximizing threshold of 40 (Fig. 2), classifying 85% of the major invaders as high risk and 93% of non-invaders (including 84% of minor invaders) as low risk. Setting the threshold at the intermediate score of 31 results in >95% of both major and non-invaders correctly classified, with 63% of minor invaders classed as high risk. Selection of the threshold for implementation may vary depending on the definition of acceptable risk associated with minor invaders determined by the user (Figs. 2, 3).

The AWRA was developed with empirically determined score thresholds that separated species with low risk of becoming invasive from those with high risk; species with scores between these thresholds require further evaluation [18]. This approach reduces misclassification but delays the risk assessment decision for some species. However, the USAqWRA appears to have sufficient performance for policy application without referring species for additional testing.

Both using single or dual (i.e., referring species with intermediate scores for further testing) thresholds resulted in good assessment of the 20 validation species (Table S5). High accuracy was achieved when thresholds of either 29 or 31 were used: in that case, all invaders and 90% of the non-invaders were correctly assessed. All species were correctly classified when the two threshold system (scores 31–39 require further evaluation) was used, although 20% (2) of the invaders and 10% (1) of the non-invaders would require additional evaluation.

We used 30 years as the time-frame over which species are likely to become naturalized based on earlier work that found no correlation between year of introduction and prediction of invasiveness in many of these same species [26]. However, a precautionary approach suggests that species introduced more recently that have scores near the thresholds (e.g., *Hydrocotyle vulgaris*, score = 33) may be incipient invaders that warrant management or close monitoring.

Identification of the true accuracy of this tool would require data on the real population proportions of minor and major invaders [39]. Ideally, it would also be calibrated to reduce overall damage by including data about the costs of false positives (i.e., non-invader restricted from import) and false negatives (i.e., invader allowed for import) [23]. Although our dataset included more non-invaders than invaders, the true proportion of non-invaders introduced to the US is likely higher [8]. However, the high calculated AUC values, which are independent of the proportion in either category [41], shows that the USAqWRA is a high performance tool. While sufficient economic data are not available for inclusion in the analysis, the cost of a false negative (i.e., harmful invader allowed in) is likely to be higher than the cost of a false positive (i.e., non-invader kept out of trade) [39]. This difference suggests that a cost-sensitive approach would support the more precautionary lower threshold identified here.

Our intent was to examine whether this tool would be useful for pre-border preventative screening of species imports. As a result, the conclusions presented here are based at the scale, and incorporate the full range of environmental conditions, of the US. Accuracy is unlikely to be maintained at regional or state scales if species intolerant of growing conditions are assessed as if they might become regionally invasive. For example, the US test includes temperate to tropical climates. Use at the regional scale would require implementation of a pre-screening system for immediately excluding species from assessment if they are not tolerant of environmental conditions within that geography. Regional testing would be required to identify whether the empirically derived thresholds identified here are appropriate for different scales and environments.

The USAqWRA, like the NZAqWRA [27], may be useful for prioritizing the management of established non-native aquatic plant species. Risk assessment results would be combined with data on the feasibility and cost of long-term control success, and the vulnerability and irreplaceability (*sensu* [43]) of the biodiversity or economic assets threatened by the existing invader. If a new species were discovered in the US, however, the USAqWRA could be used to assess whether the risk of spread and impact is sufficiently high to justify a rapid response to eradicate the infestation, or whether risk is low and management resources would be better directed elsewhere [31].

Additionally, many species act as ‘sleeper weeds’ (*sensu* [44]), perhaps remaining as minor invaders for decades before they become serious invaders. For example, *Hydrocleys nymphoides* (score = 46) has been present for over a century [33] and is currently a minor invader in Texas and Florida [45]. The USAqWRA score reflects the invasive behavior of this species in New Zealand and Australia [46] and, without evidence of an effective natural mechanism controlling populations in the US, suggests that it might be prudent to implement management efforts now, to avoid probable future impacts.

If the intended use of the tool is to inform management decisions for established species, data from within the region of interest should be included. For example, the only location in which *Luziola subintegra* is known to naturalize and become the dominant species is in Florida [47]. If assessed for the US for predictive purposes, we would not use that information and would conclude that this species has a low probability of being invasive. However, incorporating the data from Florida results in a score of 37 (Table S5), suggesting that precautionary management may be prudent.

Species that scored 60 or above in our test were all classified as major invaders. If scores are used to guide management decisions, species in this score range should be prioritized for control unless their distribution and potential for treatment suggest that management is so costly that the resources would better be allocated elsewhere. Additionally, management of species that are currently minor invaders in the US but are highly invasive in other countries should be considered, since invasiveness anywhere outside the native range is a well-documented predictor of the probability a species will become invasive (e.g., [48], [49]).

Climate tolerance coupled with current distribution may also indicate species that require immediate management. If the native or introduced range of a species includes multiple US Hardiness Zones (e.g., 1–11), but the species only occupies a few of those zones, control priority should be further evaluated. For example, *Cyperus serotinus* occupies Zones 4–11 outside of the US, but is only recorded in Zones 6–7 in the US [37]. The broad climate tolerance of this species suggests the potential for a substantial range increase in the US.

Overall, our results suggest that the USAqWRA is appropriate for prevention decisions and may have additional utility for prioritizing management efforts. Accuracy of current risk assessment systems used in the US and elsewhere should be compared against the USAqWRA for aquatic plant species. Regardless of the risk assessment system, the high accuracy with which we can distinguish non-invaders from harmful invaders at the US scale suggests that a more pro-active prevention system would both be feasible and economically beneficial [23]. Further, like the AWRA [25], this tool should be tested for accuracy in additional geographies.

## Supporting Information

Table S1 **Comparison of questions and scoring between the New Zealand Aquatic Weed Risk Assessment** [**3]**, [28], [**29**] **and the USAqWRA used for the analyses described.**
(DOC)

Table S2 **Non-native species present in the US for over 30 years that were assessed in the development of the USAqWRA system.**
(DOC)

Table S3 **Ten invasive and ten non-invasive species assessed for USAqWRA system validation.**
(DOC)

Table S4 ***A priori*** **classification for test species based upon their status in the US and predicted invasiveness risk level using the USAqWRA system.**
(DOC)

Table S5 **USAqWRA system results for the 20 validation species.**
(DOC)
