# Supplementary material for: Weed Risk Assessment for Aquatic Plants: Modification of a New Zealand System for the United States
Source: PLoS One. 2012 Jul 13;7(7):e40031. doi: 10.1371/journal.pone.0040031 (PMC3396638; doi:10.1371/journal.pone.0040031)
Supplement: Table S1 — Comparison of questions and scoring between the New Zealand Aquatic Weed Risk Assessment [3] , [28], [29] and the USAqWRA used for the analyses described. (DOC) [file pone.0040031.s001.doc]

Table S1. Comparison of questions and scoring between the New Zealand Aquatic Weed Risk Assessment (NZAqWRA) [3], [28], [29] and the USAqWRA used for the analyses described herein.

| **Question - NZAqWRA** | **Question - USAqWRA** | **Score and guidance - NZAqWRA** | **Score and guidance – USAqWRA^1^** |
| --- | --- | --- | --- |
| Temperature tolerance (1.1) | Temperature tolerance (1.1) | **(0-3)** Maximum if frost tolerant, 2 if growth checked by winter temps, 1 if dies off over winter, 0 if killed over winter. | **(0-3)** Score 3 if maintains photosynthetic tissue and summer growth form throughout winter, 2 if dies back to tuber/bulb/rhizome (or similar structure) during winter, 1 if adult plants completely die but viable seeds remain. Use a climate matching tool if direct evidence is not available. Default = 1 for annual species. |
| Range of habitat (1.2) | Range of habitat (1.2) | **(1-3)** Maximum if able to grow from water to dry land, 2 if water to wetland, or from shallow to deep (>5 m) water, 1 narrow range. | **(1-3)** Score 3 if able to grow from water to dry land, 2 if water to wetland, or from shallow to deep (>5 m) water, 1 narrow range. Default = 1 if no information is available; 2 for free-floating plants, unless more information is available. |
| Water/substrate type (1.3) | Water/substrate type tolerance (1.3) | **(1-2)** Maximum if tolerant of sandy to muddy (or peaty) substrate, or oligotrophic to eutrophic waters, 1 if restricted by either. | **(1-2)** Score 2 if tolerant of sandy to muddy (or peaty) substrate, or oligotrophic to eutrophic waters, 1 if restricted by either. Default = 1 if no information is available. |
| Water clarity (1.4) | Water clarity tolerance (1.4) | **(0-1)** Maximum if unaffected by water clarity, i.e. floating, or emergent. | **(0-1)** Score 1 if unaffected by water clarity (i.e. floating or emergent, or submergents tolerant of very low light levels, such as *Myriophyllum spicatum* and *Hydrilla verticillata*), 0 if affected by water clarity. |
| Salinity (1.5) | Salinity tolerance (1.5) | **(0-1)** Can tolerate saline conditions, or not. | **(0-1)** Score 1 if species can tolerate saline conditions, 0 if not. Habitat information can be used to determine a score of 0 if species is only found to occur in freshwater habitats. |
| N/A | pH tolerance (1.6) | *Question not included in assessment.* | **(0-1)** Score 1 if tolerant of both acidic and basic pH or no information is available, 0 if restricted to neutral, basic, or acidic pH. |
| Water level fluctuation - (1.7) | Water level fluctuation - Tolerates periodic flooding/drying (1.7) | *Question was not included in the original assessment [3], but was included in assessments developed for Australia [28] and Micronesia [29].*  **(0-3)** Maximum if able to tolerate one month dewatering. | **(0-3)** Score 3 for species which have evidence of tolerating periodic flooding/drying with a specified time period longer than 1 month (e.g., "months"; "X months", "winter flooding"), 2 for evidence of tolerance of flooding/drying over a period of days/a couple of weeks, 1 for species that die back during periods of flooding/drying, and 0 for species that do not tolerate flooding/drying. Do not score if there is no information available. |
| Lentic - rivers, streams, drains, irrigation channels (2.1) | Lentic - rivers, streams, drains, or other flowing waters, including their margins (2.1) | **(0-3)** Maximum if major weed, 2 if minor weed, 1 if present but not weedy, 0 absent. | **(0-3)** Score 3 if major weed (reaches high density and dominates plant community), 2 if minor weed (common, but rarely or never dominant), 1 if present but not weedy, 0 if absent. |
| Lotic - ponds, shallow and deep lakes (2.2) | Ponds, lakes and other standing waters, including their margins (2.2) | **(0-3)** Maximum if major weed, 2 if minor weed, 1 if present but not weedy, 0 absent. | **(0-3)** Score 3 if major weed (reaches high density and dominates plant community), 2 if minor weed (common, but rarely or never dominant), 1 if present but not weedy, 0 if absent. |
| Wetland - water margin, swamp, marsh, bog (2.3) | Swamp, marsh, bog, or other wet areas not covered by 2.1 or 2.2 (2.3) | **(0-3)** Maximum if major weed, 2 if minor weed, 1 if present but not weedy, 0 absent. | **(0-3)** Score 3 if major weed (reaches high density and dominates plant community), 2 if minor weed, 1 if present but not weedy, 0 if absent. |
| Establishment – Existing vegetation (2.4) | Establishment – into existing vegetation (2.4) | *Question was not included in the original assessment [3], but was included in the assessment developed for Micronesia [29].*  **(-5 - 0)** Maximum if able to invade unmodified vegetation. | **(-5, -3, 0)** Score 0 if able to invade unmodified vegetation, -3 if the species can only colonize certain types of vegetation (e.g., turf-forming shoreline vegetation), -5 if there is no evidence that the species can move into intact vegetation. Default = 0 if there is evidence of establishment, but no specific information about level of invasion into existing vegetation and/or type of vegetation being invaded. Default = -3 for species that have not naturalized outside of their native range. |
| Establishment - Disturbance (2.5) | Establishment – into disturbed vegetation (2.5) | *Question was not included in the original assessment [3], but was included in the assessment developed for Micronesia [29].*  **(0 - 5)** Maximum if able to aggressively colonise following vegetation clearance, newly constructed waterbodies or nutrient enrichment. | **(0, 1, 5)** Score 5 if able to aggressively colonize following vegetation clearance, newly constructed waterbodies or nutrient enrichment, 1 if the species grows in disturbed areas, but there is no other information, 0 if there is no evidence of establishment in disturbed areas. Information from either the native or introduced range may be used to answer this question. Default = 1 for no information. |
| Competitive ability - Within growth form, i.e. submerged, floating, emergent (3.1) | N/A^1^ | **(0-8)** e.g. Maximum *Hydrilla:Ceratophyllum:Egeria/ Lagarosiphon/Elodea/P. crispus*/native species. | N/A |
| Competitive ability - Between growth form (3.2) | Competition – between growth form (3.1) | **(0, 1, 2)** Maximum if able to completely displace another growth form, 1 if some suppression, 0 no interaction. | **(0, 1, 2)** Score 2 if species forms dense stands that are documented to displace other growth forms (submerged, floating, emergent), 1 if some suppression, 0 if no displacement. Default = 0 if species has been in the trade globally for >30 years and there is no information about the species displacing other growth forms. |
| Dispersal outside catchment by natural agents, e.g. birds, wind (4.1) | Dispersal outside catchment by natural agents (e.g. birds, wind) (4.1) | **(0, 1, 3, 5)** Maximum if propagule well adapted for bird/wind distribution, 1 if propagule could be spread in bird crop. | **(0, 1, 3, 5)** Score 5 if species (including seeds, rhizomes, fragments etc.) well adapted, and likely to be frequently dispersed, by natural agents, 3 if transport by natural agents is possible but uncommon, 1 if propagule could be spread in bird crop, 0 if no, or extremely low, likelihood of dispersal by natural agents (e.g., *Hydrilla* is scored 1 because its turions can survive passage through duck guts, an agent of dispersal, but this is believed to happen rarely). |
| Dispersal outside catchment by accidental human activity, e.g. drainage machinery, boat trailers, eel nets (4.2) | Dispersal outside catchment by accidental human activity (4.2) | **(0-3)** Maximum if spread by 3 methods, etc. | **(1, 2, 3)** Score 3 if major pathway, seeds/fragments adapted for easy transportation (e.g., via boat/trailer, fishing gear), 2 if the species is a floating plant or a macrophyte, but no explicit mention of high spread in the literature, 1 not mentioned, not likely to be spread by human activity based on growth form and life history. Default = 1 if no information is available. |
| Dispersal outside catchment by deliberate introduction (4.3) | Dispersal outside catchment by deliberate introduction (4.3) | **(0-1)** Maximum if attractive to humans (ornamental fishpond or aquarium). If species is not used or no information exists, it should be scored a 0. | **(0-1)** Score 1 if species is desirable to humans (e.g., or used for medicinal, food, ornamental, restoration, etc. purposes in the U.S. or elsewhere). If species is not used or no information exists, score should be 0. |
| Effective spread within waterbody/ catchment (4.4) | Effective spread within waterbody/ catchment (4.4) | **(0-1)** Maximum if effective spread within waterbody by seed, or plant fragments. | **(0-1)** Score 1 for extensive spread within a waterbody or among waterbodies, 0 for no spread. Occurrence along streams or riverbanks or in rivers can be used as evidence, as well as evidence of water dispersal. Do not answer if no information is available. |
| Maturation rate (5.1) | Generation time (time between germination of an individual and the production of living offspring, not seeds or other dormant structures) (5.1) | **(1-3)** Includes growth rate and time to maturity under ideal conditions. | **(1, 2, 3)** Score 3 if rapid (reproduction in first year and >1 generation/year), 2 if annual or produces one generation every year including the first year, 1 if not reproductively mature in the first year. Default = 1 if no information is available. |
| Seeding ability - Quantity (6.1) | Seeding ability - Quantity (6.1) | **(0-3)** Maximum if >1000 seeds/plant, 2 100-1000, 1 <100, 0 nil. | **(0-3)** Score 3 if >1000 seeds/plant/year, 2 100-1000, 1 <100 and/or evidence that seed are produced (in native or introduced range), 0 if seed not produced. |
| Seeding ability – Viability/ persistence (6.2) | Seeding ability - Viability/ persistence (6.2) | **(0-2)** Maximum if high viability for several years, 1 low viability. | **(0-2)** Score 2 if highly viable for >3 years, 1 low viability or evidence of seed production with no information on viability, 0 no viable seeds. |
| Cloning ability (7.1) | Vegetative reproduction (7.1) | **(0-5)** Maximum for far-reaching rhizomes/stolons/fragmentation capable of forming new colonies, 3 for rhizome/stolons, 1 for clump forming, 0 no vegetative spread. | **(0, 1, 3, 5)** Score 5 for naturally fragmenting from rhizomes, stolons, or other vegetative growth into tissue capable of producing new colonies (e.g., *Egeria* *densa*), 3 if produces rhizomes/stolons, but there is no other information about the formation of new colonies elsewhere, 1 for clump-forming by vegetative spread, 0 for no vegetative spread. |
| Obstruction – Physical – water use (recreation) (8.1) | Physical-water use, recreation (8.1) | **(0-2)** Maximum for major nuisance, 1 minor nuisance. | **(0-2)** Score 2 for major nuisance, 1 for minor nuisance. Default = 0 if the species has not naturalized outside of its native range. If there is a reasonable amount of information about the species and it has naturalized outside of its native range, default = 0. |
| Obstruction – Physical – access (8.2) | Physical – access (8.2) | **(0-2)** Maximum for major nuisance, 1 minor nuisance. | **(0-2)** Score 2 for major nuisance, 1 for minor nuisance. Default = 0 if the species has not naturalized outside of its native range. If there is a reasonable amount of information about the species and it has naturalized outside of its native range, default = 0. |
| Obstruction – Physical – water flow, power generation (8.3) | Physical - water flow, power generation (8.3) | **(0-2)** Maximum for major nuisance, 1 minor nuisance. | **(0-2)** Score 2 for major nuisance, 1 for minor nuisance. Default = 0 if the species has not naturalized outside of its native range. If there is a reasonable amount of information about the species and it has naturalized outside of its native range, default = 0. |
| Obstruction – Physical – irrigation, flood control (8.4) | Physical - irrigation, flood control (8.4) | **(0-2)** Maximum for major nuisance, 1 minor nuisance. | **(0-2)** Score 2 for major nuisance, 1 for minor nuisance. Default = 0 if the species has not naturalized outside of its native range. If there is a reasonable amount of information about the species and it has naturalized outside of its native range, default = 0. |
| Aesthetic – visual, olfactory (8.5) | Aesthetic - visual, olfactory (8.5) | **(0-2)** Maximum for both visual and smell problems, 1 either. | **(0-2)** Score 2 for both visual and odor problems, 1 either, 0 neither or no mention of these impacts. Surface matting of macrophytes scores 1 for visual impact. |
| Damage to natural areas - Reduce biodiversity (9.1) | Reduces biodiversity (9.1) | **(0, 1, 3, 5)** Maximum for forming monospecific stands, reducing score for lessening impact. | **(0, 1, 3, 5)** Score 5 for extensive monospecific stands, 3 for species that become dominant, 1 for small monospecific stands, and 0 if species does not become dominant over other species. Default = 0 for this question if species has been in the trade globally for >30 years and no information is found or if the species is not naturalized outside of its native range. |
| Damage to natural areas - Reduce water quality (9.2) | Reduces water quality (9.2) | **(0-3)** Maximum for major impacts especially deoxygenation. | **(0, 1, 3)** Score 3 if evidence that this species causes deoxygenation (e.g., through extensive growth in shallow water) or other water quality loss (e.g., loss of water clarity because of high decomposition rates continuously during the growing season), 1 if deoxygenation or other water quality loss is likely based on seasonal growth cycles (e.g., macrophyte that gets to high density and dies off at end of summer), 0 otherwise. Default = 0 for this question if species has been in the trade globally for >30 years and no information is found or if the species is not naturalized outside of its native range. |
| Damage to natural areas - Negatively affect physical processes (9.3) | Negatively affect physical processes (9.3) | **(0-2)** Maximum for major effects on substrate stability, hydrology (flooding). | **(0, 2)** Score 2 if species alters hydrology (e.g., increases the chance of flooding) or substrate stability (e.g., increases amount of sediment erosion or deposition), or other physical processes, 0 if the species has no history of modifying physical processes. Default = 0 for this question if species has been in the trade globally for >30 years and no information is found or if the species is not naturalized outside of its native range. |
| Other undesirable traits - Health impairment, e.g. drowning, poisonous, sharp leaf edges, mosquito breeding habitat (10.1) | Human health impairment (e.g. drowning, poisonous, mosquito habitat) (10.1) | **(0-2)** Maximum for 2 or more effects. | **(0-2)** Score 1 for one effect, 2 for 2 or more effects. |
| Other undesirable traits - Weed of agriculture (10.2) | Weed of agriculture, including crops, livestock and aquaculture (10.2) | **(0-1)** Maximum if a problem land weed. | **(0-1)** Score 1 if a problem agricultural weed, 0 if no evidence that it is an agricultural weed, or if evidence states that species is in agricultural areas but not problematic. |
| Extent of suitable habitat (11.1) | N/A^2^ | Available habitat present in NZ scored out of 10, amount of available habitat not occupied scored as a fraction, e.g. alligator weed 4/6 (scores 4), raupo 0/10 (scores 0), hydrilla 9/10 (scores 9). | N/A |
| Resistance to management - Ease of implementation (12.1) | Management - Ease of management implementation (11.1) | **(0-2)** Maximum if accessibility to weed is difficult, e.g. dense tall impenetrable growths. | **(0-2)** Score 2 if accessibility to weed is difficult, e.g. dense tall impenetrable growths or growing in habitats that are difficult to access by roads or waterways (e.g., swamps). For species that have naturalized outside of their native range, default = 0-2 based upon evidence about habitat and/or growth form if there is no direct evidence from the literature. Default = 0 if species has not naturalized outside of its native range and has been in the trade globally for >30 years. |
| Resistance to management - Recognition of problem (12.2) | Management - Recognition of management problem (11.2) | **(0-1)** Maximum if difficult to assess weed, e.g., submerged. | **(0-1)** Score 1 if difficult to assess weed, e.g., submerged; looks like another species. For species that have naturalized outside of their native range, default to a score between 0-1 based upon growth form evidence if there is no direct evidence from the literature. Default = 0 if species has not naturalized outside of its native range and has been in the trade globally for >30 years. |
| Resistance to management - Scope of control methods (12.3) | Management - Scope of control methods (11.3) | **(0-2)** Maximum if no control method, 1 if only one control option. | **(0, 1, 2)** Score 2 if no control method, 1 if only one control option. If species has naturalized outside of its native range, and there is no direct evidence for either 11.1 or 11.2, do not answer if there is no information. If there is direct evidence for 11.1 and/or 11.2, default to 0 if there is no information for this question. Default = 0 if species has not naturalized outside of its native range and has been in the trade globally for >30 years. |
| Resistance to management - Suitability (12.4) | Management - Control method suitability (11.4) | **(0-1)** Maximum if control method not always acceptable, e.g., grass carp, unregistered herbicide. | **(0-1)** Score 1 if control method not always acceptable, e.g., grass carp, unregistered herbicide. If species has naturalized outside of its native range, and there is no direct evidence for either 11.1 or 11.2, do not answer if there is no information. If there is direct evidence for 11.1 and/or 11.2, default to 0 if there is no information for this question. Default = 0 if species has not naturalized outside of its native range and has been in the trade globally for >30 years. |
| Resistance to management - Effectiveness (12.5) | Management - Effectiveness of control (11.5) | **(0-2)** Maximum if ineffective, 1 if partial control. | **(0, 1, 2)** Score 2 if ineffective, 1 if partial control. If species has naturalized outside of its native range, and there is no direct evidence for either 11.1 or 11.2, do not answer if there is no information. If there is direct evidence for 11.1 and/or 11.2, default to 0 if there is no information for this question. Default = 0 if species has not naturalized outside of its native range and has been in the trade globally for >30 years. |
| Resistance to management - Duration of control (12.6) | Management - Duration of control (11.6) | **(0-2)** Maximum if no control, 1 if control for 3+ months. | **(0, 1, 2)** Score 2 if no control, 1 if control for 3+ months. If species has naturalized outside of its native range, and there is no direct evidence for either 11.1 or 11.2, do not answer if there is no information. If there is direct evidence for 11.1 and/or 11.2, default to 0 if there is no information for this question. Default = 0 if species has not naturalized outside of its native range and has been in the trade globally for >30 years. |
| Problem in other countries (13.1) | Problem in other countries (12.1) | **(0 - 5)** Maximum if widespread problem weed in other temperate countries, 4 only problem in some temperate countries, 3 if adventive, not weedy in other temperate countries, 2 if tropical weed, 1 if adventive, but not weedy in tropics, 0 not adventive elsewhere. | **(0, 1, 3, 4, 5)** Score 5 if species has been reported to be a widespread problem (i.e., a harmful weed in many other countries), 4 if species has been reported to be a harmful weed in 5 or fewer countries, 3 if species has been reported to be a widespread adventive (but not a harmful weed) in many other countries, 1 if species has been reported to be adventive in 5 or fewer countries, 0 if not adventive elsewhere. |

^1^A maximum of five questions may be left unanswered for completion of the USAqWRA. For this purpose, no response for any or all of Q. 2.1-3 or Q. 11.1-6 should be counted as one unanswered question each (see text).

^2^ Removed from assessment (see text).
