# Supplementary material for: Weed Risk Assessment for Aquatic Plants: Modification of a New Zealand System for the United States
Source: PLoS One. 2012 Jul 13;7(7):e40031. doi: 10.1371/journal.pone.0040031 (PMC3396638; doi:10.1371/journal.pone.0040031)
Supplement: Table S2 — Non-native species present in the US for over 30 years that were assessed in the development of the USAqWRA system. (DOC) [file pone.0040031.s002.doc]

**Table S2.** Non-native species present in the U.S. for over 30 years that were assessed in the development of the USAqWRA model.

| **Scientific Name** | **Common name** | **Family** | **Growth Form** | **U.S. Intro Date^1^** | **Global Intro Date^1^** |
| --- | --- | --- | --- | --- | --- |
| *Acorus calamus* L. | Sweet-flag | Acoraceae | Erect emergent | 1897 | 1897 |
| *Acorus gramineus* Sol. ex Aiton | Grass-leaf sweet-flag | Acoraceae | Sprawling emergent^2^ | 1897 | 1897 |
| *Aldrovanda vesiculosa* L. | Waterwheel plant | Droseraceae | Free-floating | 1967 | 1947 |
| *Alisma plantago-aquatica* L. | Water-plantain | Alismataceae | Erect emergent | 1897 | 1897 |
| *Alternanthera philoxeroides* (Mart.) Griseb. | Alligator-weed | Amaranthaceae | Sprawling emergent | 1928 | 1928 |
| *Alternanthera sessilis* (L.) R. Br. ex DC. | Sessile joyweed | Amaranthaceae | Sprawling emergent | 1945 | 1945 |
| *Ammannia senegalensis* Lam.^3^ | Red ammannia | Lythraceae | Erect emergent; submerged^2^ | 1959 | 1959 |
| *Anubias afzelii* Schott | Afzeli anubias | Araceae | Amphibious submerged^2^ | 1976 | 1960 |
| *Anubias barteri* Schott | Giant anubias | Araceae | Amphibious submerged^2^ | 1959 | 1959 |
| *Anubias barteri* Schott var. *glabra* N.E. Br. | Anubias | Araceae | Amphibious submerged^2^ | 1959 | 1959 |
| *Aponogeton crispus* Thunb. | Ruffled sword plant | Aponogetonaceae | Submerged^2^ | 1967 | 1932 |
| *Aponogeton distachyos* L. f. | Cape-pondweed | Aponogetonaceae | Water lily type (attached-floating) | 1897 | 1897 |
| *Aponogeton madagascariensis* (Mirb.) H. Bruggen | Laceleaf | Aponogetonaceae | Erect emergent; submerged^2^ | 1855 | 1855 |
| *Aponogeton natans* (L.) Engl. & Krause | Floating lace plant | Aponogetonaceae | Erect emergent; submerged^2^ | 1967 | 1947 |
| *Aponogeton ulvaceus* Baker | Compact aponogeton | Aponogetonaceae | Submerged | 1967 | 1947 |
| *Bolbitis heteroclita* (C. Presl) Ching | Asian water fern | Lomariopsidaceae | Amphibious submerged^2^ | 1959 | 1959 |
| *Bolbitis heudelotii* (Bory ex Fée) Alston | African water fern | Lomariopsidaceae | Amphibious submerged^2^ | 1977 | 1960 |
| *Bolboschoenus glaucus* (Lam.) S.G. Sm. (=*Schoenoplectus glaucus* (Lam.) Kartesz) | Tuberous bulrush | Cyperaceae | Erect emergent | 1923 | 1923 |
| *Butomus umbellatus* L. | Flowering-rush | Butomaceae | Erect emergent | 1897 | 1897 |
| *Callitriche stagnalis* Scop. emend. Kutz | European water-starwort | Callitrichaceae | Attached-floating; amphibious submerged; sprawling emergent | 1861 | 1861 |
| *Canna × generalis* L.H. Bailey & E.Z. Bailey | Common garden canna | Cannaceae | Erect emergent | 1930 | 1930 |
| *Canna indica* L. | Edible canna | Cannaceae | Erect emergent | 1947 | 1947 |
| *Cardamine lyrata* Bunge | Chinese-ivy | Brassicaceae | Amphibious submerged; sprawling emergent^2^ | 1967 | 1947 |
| *Ceratophyllum muricatum* subsp. *australe* (Griseb.) Les^3^ | Prickly hornwort | Ceratophyllaceae | Submerged | 1950 | 1950 |
| *Ceratophyllum submersum* L.^3^ | Soft hornwort | Ceratophyllaceae | Submerged | 1967 | 1947 |
| *Colocasia esculenta* (L.) Schott | Taro | Araceae | Erect emergent | 1905 | 1905 |
| *Colysis pteropus* (Blume) Bosman (=*Microsorium pteropus* (Blume) Copel.) | Java fern | Polypodiaceae | Amphibious submerged^2^ | 1929 | 1929 |
| *Crinum erubescens* Aiton | Swamp lily | Amaryllidaceae | Erect emergent | 1949 | 1949 |
| *Cryptocoryne × willisii* Reitz | Willis' cryptocoryne | Araceae | Amphibious submerged^2^ | 1949 | 1949 |
| *Cryptocoryne ciliata* (Roxb.) Fisch. ex Wydl. | Ciliata | Araceae | Amphibious submerged^2^ | 1967 | 1947 |
| *Cryptocoryne cordata* Griff. | Cryptocoryne | Araceae | Amphibious submerged^2^ | 1967 | 1947 |
| *Cryptocoryne retrospiralis* (Roxb.) Fisch. Ex Wydl. (=*Cryptocoryne crispatula* Engl.) | Balansae crypto | Araceae | Amphibious submerged^2^ | 1967 | 1947 |
| *Cyperus difformis* L. | Small-flower umbrella-plant | Cyperaceae | Erect emergent | 1934 | 1934 |
| *Cyperus involucratus* Rottb. | Umbrella sedge | Cyperaceae | Erect emergent | 1909 | 1909 |
| *Cyperus longus* L. | Sweet cyperus | Cyperaceae | Erect emergent | 1901 | 1901 |
| *Cyperus prolifer* Lam. | Dwarf papyrus | Cyperaceae | Erect emergent | 1947 | 1947 |
| *Cyperus serotinus* Rottb. | Tidal marsh flat sedge | Cyperaceae | Erect emergent | 1935 | 1935 |
| *Echinodorus martii* Micheli (=*Echinodorus major* (Micheli) Rataj) | Ruffled Amazon sword | Alismataceae | Erect emergent; submerged^2^ | 1967 | 1947 |
| *Echinodorus palaefolius* (Nees & Mart.) J.F. Macbr. | Mexican sword-plant | Alismataceae | Erect emergent^2^ | 1959 | 1959 |
| *Echinodorus paniculatus* Micheli | Amazon sword-plant | Alismataceae | Erect emergent; submerged^2^ | 1967 | 1947 |
| *Echinodorus uruguayensis* Arechav. | Uruguay Amazon sword | Alismataceae | Erect emergent; submerged^2^ | 1950 | 1950 |
| *Egeria densa* Planch. | Brazilian elodea | Hydrocharitaceae | Submerged | 1893 | 1893 |
| *Eichhornia azurea* (Sw.) Kunth | Anchored water-hyacinth | Pontederiaceae | Attached-floating; obligate submerged; water-lily type | 1897 | 1897 |
| *Eichhornia crassipes* (Mart.) Solms | Water-hyacinth | Pontederiaceae | Free-floating | 1897 | 1897 |
| *Eichhornia paniculata* (Spreng.) Solms | Brazilian water hyacinth | Pontederiaceae | Erect emergent | 1913 | 1913 |
| *Elatine macropoda* Guss. | Southern waterwort | Elatinaceae | Submerged | 1967 | 1947 |
| *Eriophorum latifolium* Hoppe | Grey cotton-grass | Cyperaceae | Erect emergent | 1949 | 1949 |
| *Euryale ferox* Salisb. ex K.D. Koenig & Sims | Gorgon | Nymphaeaceae | Water lily type (attached-floating) | 1897 | 1897 |
| *Glyceria fluitans* (L.) R. Br. | Floating manna grass | Poaceae | Sprawling emergent | 1930 | 1930 |
| *Glyceria maxima* (Hartm.) Holmb. | Reed sweet grass | Poaceae | Sprawling emergent | 1940 | 1940 |
| *Gratiola officinalis* L. | Gratiola | Plantaginaceae | Erect emergent; sprawling emergent | 1930 | 1930 |
| *Gratiola peruviana* L. | Austral brooklime | Plantaginaceae | Erect emergent | 1833 | 1833 |
| *Hesperantha coccinea* (Backh. & Harv.) Goldblatt & J.C. Manning (=*Schizostylis coccinea* Backh. & Harv.) | River-lily | Iridaceae | Erect emergent | 1930 | 1901 |
| *Heteranthera zosterifolia* Mart. | Stargrass | Pontederiaceae | Erect emergent; submerged^2^ | 1967 | 1932 |
| *Hottonia palustris* L. | Water-violet | Primulaceae | Submerged | 1897 | 1901 |
| *Houttuynia cordata* Thunb. | Chameleon-plant | Saururaceae | Erect emergent | 1976 | 1947 |
| *Hydrilla verticillata* (L. f.) Royle | Hydrilla | Hydrocharitaceae | Submerged | 1959 | 1959 |
| *Hydrocharis morsus-ranae* L. | European frog's-bit | Hydrocharitaceae | Free-floating | 1897 | 1897 |
| *Hydrocleys nymphoides* (Willd.) Buchenau | Water-poppy | Limnocharitaceae | Attached-floating | 1897 | 1897 |
| *Hydrocotyle vulgaris* L. | Marsh pennywort | Araliaceae | Sprawling emergent^2^ | 1967 | 1947 |
| *Hydrostemma longifolium* (Wall.) Mabb. (=*Barclaya longifolia* Wall.) | Orchid lily | Nymphaeaceae | Submerged | 1967 | 1958 |
| *Hygrophila corymbosa* Lindau^3^ | Giant hygrophila | Acanthaceae | Erect emergent^2^ | 1917 | 1917 |
| *Hygrophila difformis* (L.f.) Blume | Water-wisteria | Acanthaceae | Erect emergent; submerged^2^ | 1967 | 1957 |
| *Hygrophila polysperma* (Roxb.) T. Anderson | Indian swampweed | Acanthaceae | Erect emergent^2^ | 1947 | 1947 |
| *Hymenachne amplexicaulis* (Rudge) Nees | West Indian marsh grass | Poaceae | Sprawling emergent | 1968 | 1968 |
| *Ipomoea aquatica* Forssk. | Chinese water-spinach | Convolvulaceae | Free-floating; sprawling emergent | 1949 | 1949 |
| *Iris ensata* Thunb. | Japanese water iris | Iridaceae | Erect emergent | 1897 | 1897 |
| *Iris pseudacorus* L. | Yellow-flag iris | Iridaceae | Erect emergent | 1868 | 1868 |
| *Landoltia punctata* (G. Mey.) Les & D.J. Crawford | Dotted duckmeat | Araceae | Free-floating | 1930 | 1930 |
| *Lasia spinosa* (L.) Thwaites | Lasia | Araceae | Erect emergent | 1959 | 1959 |
| *Lilaeopsis novae-zelandiae* A.W. Hill | Micro sword | Apiaceae | Amphibious submerged^2^ | 1979 | 1979 |
| *Limnocharis flava* (L.) Buchenau | Sawah-flower rush | Limnocharitaceae | Free-floating | 1934 | 1934 |
| *Limnophila indica* (L.) Druce | Ambulia | Limnocharitaceae | Amphibious submerged^2^ | 1967 | 1947 |
| *Limnophila sessiliflora* (Vahl) Blume | Ambulia | Limnocharitaceae | Amphibious submerged^2^ | 1961 | 1947 |
| *Ludwigia adscendens* (L.) H. Hara | Water-primrose | Onagraceae | Sprawling emergent | 1979 | 1947 |
| *Ludwigia helminthorrhiza* (Mart.) H. Hara | Rattlebox | Onagraceae | Free-floating^2^ | 1976 | 1917 |
| *Ludwigia peruviana* (L.) H. Hara | Peruvian primrosebush | Onagraceae | Sprawling emergent^2^ | 1929 | 1929 |
| *Lythrum salicaria* L. | Purple loosestrife | Lythraceae | Erect emergent | 1831 | 1831 |
| *Marsilea drummondii* A. Braun | Common nardoo | Marsileaceae | Attached-floating^2^ | 1949 | 1947 |
| *Marsilea quadrifolia* L. | European water-clover | Marsileaceae | Attached-floating^2^ | 1860 | 1860 |
| *Mentha aquatica* L. | Water mint | Lamiaceae | Sprawling emergent^2^ | 1933 | 1898 |
| *Murdannia keisak* (Hassk.) Hand.-Mazz. | Marsh dewflower | Commelinaceae | Sprawling emergent^2^ | 1935 | 1935 |
| *Myosotis scorpioides* L. | Forget-me-not | Boraginaceae | Sprawling emergent | 1886 | 1886 |
| *Myriophyllum aquaticum* (Vell.) Verdc. | Parrot's-feather | Haloragaceae | Sprawling emergent^2^ | 1890 | 1890 |
| *Myriophyllum spicatum* L. | Eurasian water-milfoil | Haloragaceae | Submerged | 1905 | 1905 |
| *Najas minor* All. | Brittle naiad | Najadaceae | Submerged | 1932 | 1932 |
| *Nasturtium microphyllum* Boenn. ex Rchb. | One-row watercress | Brassicaceae | Attached-floating; sprawling emergent | 1946 | 1946 |
| *Nasturtium officinale* R. Br. (=*Rorippa nasturtium-aquaticum* (L.) Hayek) | Watercress | Brassicaceae | Sprawling emergent | 1831 | 1831 |
| *Nechamandra alternifolia* (Roxb.) Thwaites | Nechamandra | Hydrocharitaceae | Submerged | 1970 | 1947 |
| *Nelumbo nucifera* Gaertn. | East Indian lotus | Nelumbonaceae | Water lily type (attached-floating) | 1897 | 1897 |
| *Nymphaea × daubenyana* W.T. Baxter ex Daubeny | Dauben's waterlily | Nymphaeaceae | Water lily type (attached-floating) | 1934 | 1932 |
| *Nymphaea candida* C. Presl | Hardy waterlily | Nymphaeaceae | Water lily type (attached-floating) | 1897 | 1897 |
| *Nymphaea capensis* Thunb. var*. zanzibariensis* Conard | Cape blue water-lily | Nymphaeaceae | Water lily type (attached-floating) | 1897 | 1897 |
| *Nymphaea colorata* Peter. | Blue pygmy | Nymphaeaceae | Water lily type (attached-floating) | 1940 | 1947 |
| *Nymphaea lotus* L. | Egyptian lotus | Nymphaeaceae | Water lily type (attached-floating) | 1897 | 1897 |
| *Nymphoides crenata* (F. Muell.) Kuntze | Wavy marshwort | Menyanthaceae | Water lily type (attached-floating) | 1917 | 1917 |
| *Nymphoides indica* (L.) Kuntze | Water-snowflake | Menyanthaceae | Attached-floating; free-floating | 1897 | 1897 |
| *Nymphoides peltata* (S.G. Gmel.) Kuntze | Yellow floating-heart | Menyanthaceae | Attached-floating | 1863 | 1863 |
| *Oenanthe aquatica* (L.) Poir. | Fine-leaf water-dropwort | Apiaceae | Sprawling emergent | 1836 | 1836 |
| *Ottelia alismoides* (L.) Pers. | Duck-lettuce | Hydrocharitaceae | Submerged | 1939 | 1939 |
| *Panicum repens* L. | Torpedograss | Poaceae | Sprawling emergent | 1891 | 1891 |
| *Persicaria hydropiper* (L.) Opiz (=*Polygonum hydropiper* L.) | Marsh-pepper smartweed | Polygonaceae | Erect emergent | 1893 | 1893 |
| *Philydrum lanuginosum* Banks & Sol. ex Gaertn. | Frogmouth | Philydraceae | Sprawling emergent | 1847 | 1847 |
| *Pistia stratiotes* L. | Water-lettuce | Araceae | Free-floating | 1897 | 1897 |
| *Potamogeton crispus* L. | Curly-leaf pondweed | Potamogetonaceae | Submerged | 1860 | 1860 |
| *Potamogeton gayii* A. Benn. | Slender pondweed | Potamogetonaceae | Submerged | 1967 | 1960 |
| *Potamogeton wrightii* Morong | Potamogeton | Potamogetonaceae | Submerged | 1967 | 1947 |
| *Ranunculus lingua* L. | Greater spearwort | Ranunculaceae | Erect emergent | 1949 | 1901 |
| *Regnellidium diphyllum* Lindm. | Two-Leaf water clover | Marsileaceae | Water lily type (attached-floating) | 1941 | 1941 |
| *Ricciocarpos natans* (L.) Corda | Purple-fringed riccia | Ricciaceae | Free-floating; sprawling emergent | 1893 | 1893 |
| *Rotala rotundifolia* (Buch.-Ham. ex Roxb.) Koehne | Roundleaf toothcup | Lythraceae | Amphibious submerged^2^ | 1967 | 1960 |
| *Sagittaria sagittifolia* L. subsp. *leucopetala* (Miq.) Hartog | Chinese arrowhead | Alismataceae | Erect emergent | 1905 | 1901 |
| *Salvinia minima* Baker | Water spangles | Salviniaceae | Free-floating | 1889 | 1889 |
| *Salvinia natans* All. | Floating watermoss | Salviniaceae | Free-floating | 1897 | 1897 |
| *Saururus chinensis* (Lour.) Baill. | Chinese lizard’s tail | Saururaceae | Erect emergent | 1901 | 1901 |
| *Schoenoplectus mucronatus* (L.) Palla | Rice-field bulrush | Cyperaceae | Erect emergent | 1899 | 1899 |
| *Trapa natans* L. | European water-chestnut | Trapaceae | Free-floating | 1874 | 1874 |
| *Typha × glauca* Godr. | Cattail | Typhaceae | Erect emergent | 1950 | 1950 |
| *Typha angustifolia* L. | Narrow-leaf cattail | Typhaceae | Erect emergent | 1880 | 1880 |
| *Typha minima* Funck in Hoppe | Dwarf cattail | Typhaceae | Erect emergent | 1897 | 1897 |
| *Urochloa mutica* (Forssk.) T.Q. Nguyen | Para grass | Poaceae | Sprawling emergent | 1889 | 1889 |
| *Utricularia aurea* Lour. | Golden bladderwort | Lentibulariaceae | Free-floating^2^ | 1967 | 1947 |
| *Utricularia australis* R. Br. | Bladderwort | Lentibulariaceae | Free-floating^2^ | 1905 | 1905 |
| *Utricularia inflexa var. stellaris* (L. f.) P. Taylor (=*U. stellaris* L.f.) | Utricularia | Lentibulariaceae | Free-floating^2^ | 1967 | 1876 |
| *Vallisneria spiralis* L. | Eel-grass | Hydrocharitaceae | Submerged | 1905 | 1905 |
| *Veronica beccabunga* L. | European brooklime | Plantaginaceae | Sprawling emergent | 1876 | 1876 |
| *Vesicularia dubyana* (Müll. Hal.) Broth. | Java moss | Hypnaceae | Submerged | 1967 | 1960 |
| *Victoria amazonica* (Poepp.) J.C. Sowerby | Amazon water-lily | Nymphaeaceae | Water lily type (attached-floating) | 1897 | 1897 |
| *Victoria cruziana* A.D. Orb. | Santa Cruz water-lily | Nymphaeaceae | Water lily type (attached-floating) | 1905 | 1905 |
| *Wolffia welwitschii* Hegelm. | Pond bogmat | Araceae | Free-floating | 1974 | 1974 |

^1^The introduction dates listed represent the first date for which we found evidence that the species was in or available in the region earlier than 1980, rather than the actual date of introduction.

^2^Species are also grown as submerged plants in the aquarium trade.

^3^Species were not included in the analysis or results reported because > 5 questions were unanswered.
