# Supplementary material for: Weed Risk Assessment for Aquatic Plants: Modification of a New Zealand System for the United States
Source: PLoS One. 2012 Jul 13;7(7):e40031. doi: 10.1371/journal.pone.0040031 (PMC3396638; doi:10.1371/journal.pone.0040031)
Supplement: Table S3 — Ten invasive and ten non-invasive species assessed for USAqWRA system validation. (DOC) [file pone.0040031.s003.doc]

**Table S3**. Ten invasive and ten non-invasive species assessed for USAqWRA model validation.

| **Scientific name** | **Common name** | **Family** | **Growth Form** | **U.S. Intro Date^1^** | **Global Intro Date^1^** |
| --- | --- | --- | --- | --- | --- |
| *Azolla pinnata R.Br.* subsp. *asiatica* R.M.K. Saunders & K. Fowler | Mosquito fern | Azollaceae | Free-floating | 2007 | 1999 |
| *Glossostigma cleistanthum* W.R. Barker | Mudmat | Phrymaceae | Submerged; sprawling emergent | 1991 | 1991 |
| *Glyceria declinata* Bréb. | Waxy mannagrass | Poaceae | Sprawling emergent | 1947 | 1947 |
| *Ludwigia grandiflora* (Michx.) Greuter & Burdet subsp. *hexapetala* (Hook. & Arn.) G.L. Nesom & Kartesz | Water-primrose | Onagraceae | Erect emergent; sprawling emergent | “decades” | “decades” |
| *Luziola subintegra* Swallen | Tropical American watergrass | Poaceae | Sprawling emergent | 2007 | 2007 |
| *Marsilea minuta* L. | Small water-clover | Marsileaceae | Erect emergent; sprawling emergent | 1992 | 1992 |
| *Marsilea mutica* Mett. | Australian water-clover | Marsileaceae | Erect emergent; sprawling emergent | 1997 | 1947 |
| *Nymphoides cristata* (Roxb.) Kuntze | Crested floating heart | Menyanthaceae | Attached-floating | 1988 | 1982 |
| *Rorippa amphibia* (L.) Besser | Great yellow-cress | Brassicaceae | Erect emergent; submerged | 1950 | 1950 |
| *Salvinia molesta* D.S. Mitch. | Giant salvinia | Salviniaceae | Free-floating | 1983 | 1983 |
| *Blyxa japonica* (Miq.) Maxim. ex Asch. & Gürke | Blyxa | Hydrocharitaceae | Submerged | 1967 | 1967 |
| *Cabomba aquatica* Aubl. | Giant cabomba | Cabombaceae | Submerged | 1967 | 1932 |
| *Elatine alsinastrum* L. | Elatine | Elatinaceae | Erect emergent | 1967 | 1947 |
| *Eleocharis multicaulis* (Smith) Desv. | Many-stalked spike rush | Cyperaceae | Erect emergent | 1967 | 1967 |
| *Groenlandia densa* (L.) Fourr. | Opposite-leaved pondweed | Potamogetonaceae | Submerged | 1967 | 1947 |
| *Isoetes velata* A. Braun | Quillwort | Isoetaceae | Amphibious submerged | 1967 | 1952 |
| *Luronium natans* (L.) Raf. | Floating water-plantain | Alismataceae | Attached-floating; erect emergent; submerged | 1897 | 1897 |
| *Myriophyllum oguraense* Miki | Japanese myriophyllum | Haloragaceae | Sprawling emergent; submerged | 1967 | 1967 |
| *Nymphaea rubra* Roxb. | Indian red water-lily | Nymphaeaceae | Attached-floating | 1897 | 1897 |
| *Potamogeton lucens* L. | Shining pondweed | Potamogetonaceae | Submerged | 1967 | 1952 |

**^1^**The introduction dates listed represent the first date for which we found evidence that the species was in or available in the region earlier than 1980, rather than the actual date of introduction. All non-invaders were introduced to the U.S. prior to 1981.
