# Supplementary material for: Weed Risk Assessment for Aquatic Plants: Modification of a New Zealand System for the United States
Source: PLoS One. 2012 Jul 13;7(7):e40031. doi: 10.1371/journal.pone.0040031 (PMC3396638; doi:10.1371/journal.pone.0040031)
Supplement: Table S4 — A priori classification for test species based upon their status in the US and predicted invasiveness risk level using the USAqWRA system. (DOC) [file pone.0040031.s004.doc]

**Table S4.** *A priori* classification for test species based upon their status in the U.S. and predicted invasiveness risk level using the USAqWRA system.

| **Species name** | **U.S. *a priori* classification** | **U.S. Score** | **U.S. Risk^1^ using threshold score = 31** | **U.S. Risk^1^ using 31-39 score range for further evaluation** |
| --- | --- | --- | --- | --- |
| *Acorus calamus* | Major invader | 48 | High | High |
| *Acorus gramineus* | Non-invader | 20 | Low | Low |
| *Aldrovanda vesiculosa* | Non-invader | 27 | Low | Low |
| *Alisma plantago-aquatica* | Minor invader | 37 | High | Evaluate further |
| *Alternanthera philoxeroides* | Major invader | 75 | High | High |
| *Alternanthera sessilis* | Major invader | 44 | High | High |
| *Ammannia senegalensis* | Non-invader | -- | Unresolved^2^ | Unresolved^2^ |
| *Anubias afzelii* | Non-invader | 16 | Low | Low |
| *Anubias barteri* | Non-invader | 14 | Low | Low |
| *Anubias barteri* var. *glabra* | Non-invader | 14 | Low | Low |
| *Aponogeton crispus* | Non-invader | 14 | Low | Low |
| *Aponogeton distachyos* | Minor invader | 53 | High | High |
| *Aponogeton madagascariensis* | Non-invader | 19 | Low | Low |
| *Aponogeton natans* | Non-invader | 15 | Low | Low |
| *Aponogeton ulvaceus* | Non-invader | 13 | Low | Low |
| *Barclaya longifolia* | Non-invader | 13 | Low | Low |
| *Bolbitis heteroclitae* | Non-invader | 23 | Low | Low |
| *Bolbitis heudelotii* | Non-invader | 17 | Low | Low |
| *Butomus umbellatus* | Major invader | 62 | High | High |
| *Callitriche stagnalis* | Major invader | 38 | High | Evaluate further |
| *Canna × generalis* | Minor invader | 23 | Low | Low |
| *Canna indica* | Minor invader | 39 | High | Evaluate further |
| *Cardamine lyrata* | Non-invader | 21 | Low | Low |
| *Ceratophyllum muricatum* subsp. *australe* | Minor invader | 11 | Low | Low |
| *Ceratophyllum submersum* | Non-invader | -- | Unresolved^2^ | Unresolved^2^ |
| *Colocasia esculenta* | Major invader | 51 | High | High |
| *Colysis pteropus* (=*Microsorium pteropus*) | Non-invader | 23 | Low | Low |
| *Crinum erubescens* | Non-invader | 20 | Low | Low |
| *Cryptocoryne × willisii* | Non-invader | 11 | Low | Low |
| *Cryptocoryne ciliata* | Non-invader | 19 | Low | Low |
| *Cryptocoryne cordata* | Non-invader | 14 | Low | Low |
| *Cryptocoryne crispatula* | Non-invader | 12 | Low | Low |
| *Cyperus difformis* | Major invader | 45 | High | High |
| *Cyperus involucratus* | Major invader | 35 | High | Evaluate further |
| *Cyperus longus* | Non-invader | 19 | Low | Low |
| *Cyperus prolifer* | Minor invader | 32 | High | Evaluate further |
| *Cyperus serotinus* | Minor invader | 26 | Low | Low |
| *Echinodorus martii* (=*Echinodorus major*) | Non-invader | 11 | Low | Low |
| *Echinodorus palaefolius* | Non-invader | 12 | Low | Low |
| *Echinodorus paniculatus* | Non-invader | 16 | Low | Low |
| *Echinodorus uruguayensis* | Non-invader | 12 | Low | Low |
| *Egeria densa* | Major invader | 71 | High | High |
| *Eichhornia azurea* | Minor invader | 33 | High | Evaluate further |
| *Eichhornia crassipes* | Major invader | 81 | High | High |
| *Eichhornia paniculata* | Minor invader | 25 | Low | Low |
| *Elatine macropoda* | Non-invader | 14 | Low | Low |
| *Eriophorum latifolium* | Non-invader | 11 | Low | Low |
| *Euryale ferox* | Non-invader | 19 | Low | Low |
| *Glyceria fluitans* | Minor invader | 45 | High | High |
| *Glyceria maxima* | Major invader | 71 | High | High |
| *Gratiola officinalis* | Non-invader | 28 | Low | Low |
| *Gratiola peruviana* | Non-invader | 23 | Low | Low |
| *Hesperantha coccinea* | Non-invader | 28 | Low | Low |
| *Heteranthera zosterifolia* | Non-invader | 11 | Low | Low |
| *Hottonia palustris* | Non-invader | 30 | Low | Low |
| *Houttuynia cordata* | Minor invader | 37 | High | Evaluate further |
| *Hydrilla verticillata* | Major invader | 79 | High | High |
| *Hydrocharis morsus-ranae* | Major invader | 62 | High | High |
| *Hydrocleys nymphoides* | Minor invader | 46 | High | High |
| *Hydrocotyle vulgaris* | Non-invader | 33 | High | Evaluate further |
| *Hygrophila corymbosa* | Minor invader | -- | Unresolved^2^ | Unresolved^2^ |
| *Hygrophila difformis* | Minor invader | 29 | Low | Low |
| *Hygrophila polysperma* | Major invader | 53 | High | High |
| *Hymenachne amplexicaulis* | Major invader | 55 | High | High |
| *Ipomoea aquatica* | Major invader | 62 | High | High |
| *Iris ensata* | Minor invader | 25 | Low | Low |
| *Iris pseudacorus* | Major invader | 58 | High | High |
| *Landoltia punctata* | Major invader | 38 | High | Evaluate further |
| *Lasia spinosa* | Non-invader | 20 | Low | Low |
| *Lilaeopsis novae-zelandiae* | Non-invader | 19 | Low | Low |
| *Limnocharis flava* | Non-invader | 55 | High | High |
| *Limnophila indica* | Minor invader | 17 | Low | Low |
| *Limnophila sessiliflora* | Major invader | 33 | High | Evaluate further |
| *Ludwigia adscendens* | Non-invader | 32 | High | Evaluate further |
| *Ludwigia helminthorrhiza* | Non-invader | 16 | Low | Low |
| *Ludwigia peruviana* | Major invader | 65 | High | High |
| *Lythrum salicaria* | Major invader | 73 | High | High |
| *Marsilea drummondii* | Non-invader | 22 | Low | Low |
| *Marsilea quadrifolia* | Major invader | 61 | High | High |
| *Mentha aquatica* | Minor invader | 37 | High | Evaluate further |
| *Murdannia keisak* | Major invader | 24 | Low | Low |
| *Myosotis scorpioides* | Minor invader | 38 | High | Evaluate further |
| *Myriophyllum aquaticum* | Major invader | 75 | High | High |
| *Myriophyllum spicatum* | Major invader | 81 | High | High |
| *Najas minor* | Major invader | 66 | High | High |
| *Nasturtium microphyllum* | Minor invader | 35 | High | Evaluate further |
| *Nasturtium officinale* | Major invader | 44 | High | High |
| *Nechamandra alternifolia* | Non-invader | 20 | Low | Low |
| *Nelumbo nucifera* | Minor invader | 38 | High | Evaluate further |
| *Nymphaea × daubenyana* | Minor invader | 15 | Low | Low |
| *Nymphaea candida* | Non-invader | 17 | Low | Low |
| *Nymphaea capensis* var. *zanzibariensis* | Minor invader | 25 | Low | Low |
| *Nymphaea colorata* | Non-invader | 16 | Low | Low |
| *Nymphaea lotus* | Minor invader | 39 | High | Evaluate further |
| *Nymphoides crenata* | Non-invader | 17 | Low | Low |
| *Nymphoides indica* | Minor invader | 29 | Low | Low |
| *Nymphoides peltata* | Major invader | 74 | High | High |
| *Oenanthe aquatica* | Minor invader | 35 | High | Evaluate further |
| *Ottelia alismoides* | Minor invader | 37 | High | Evaluate further |
| *Panicum repens* | Major invader | 63 | High | High |
| *Persicaria hydropiper* (=*Polygonum hydropiper*) | Minor invader | 54 | High | High |
| *Philydrum lanuginosum* | Non-invader | 20 | Low | Low |
| *Pistia stratiotes* | Major invader | 72 | High | High |
| *Potamogeton crispus* | Major invader | 69 | High | High |
| *Potamogeton gayii* | Non-invader | 12 | Low | Low |
| *Potamogeton wrightii* | Non-invader | 19 | Low | Low |
| *Ranunculus lingua* | Non-invader | 26 | Low | Low |
| *Regnellidium diphyllum* | Non-invader | 19 | Low | Low |
| *Ricciocarpos natans* | Minor invader | 31 | High | Evaluate further |
| *Rotala rotundifolia* | Major invader | 33 | High | Evaluate further |
| *Sagittaria sagittifolia* subsp. *leucopetala* | Non-invader | 30 | Low | Low |
| *Salvinia minima* | Major invader | 70 | High | High |
| *Salvinia natans* | Minor invader | 57 | High | High |
| *Saururus chinensis* | Non-invader | 17 | Low | Low |
| *Schoenoplectus glaucus* (=*Bolboschoenus glaucus*) | Minor invader | 21 | Low | Low |
| *Schoenoplectus mucronatus* | Major invader | 40 | High | High |
| *Trapa natans* | Major invader | 66 | High | High |
| *Typha × glauca* | Major invader | 51 | High | High |
| *Typha angustifolia* | Major invader | 69 | High | High |
| *Typha minima* | Non-invader | 27 | Low | Low |
| *Urochloa mutica* | Major invader | 58 | High | High |
| *Utricularia aurea* | Non-invader | 17 | Low | Low |
| *Utricularia australis* | Non-invader | 22 | Low | Low |
| *Utricularia stellaris* | Non-invader | 20 | Low | Low |
| *Vallisneria spiralis* | Major invader | 69 | High | High |
| *Veronica beccabunga* | Minor invader | 32 | High | Evaluate further |
| *Vesicularia dubyana* | Non-invader | 12 | Low | Low |
| *Victoria amazonica* | Non-invader | 18 | Low | Low |
| *Victoria cruziana* | Non-invader | 16 | Low | Low |
| *Wolffia welwitschii* | Non-invader | 15 | Low | Low |

^1^Risk is assessed using both a single threshold score of 31 and higher identifying species with high risk of becoming invasive, and a dual threshold such that species with scores < 31 predicted to have low risk of becoming invasive, those with scores between 31 and 39 require further evaluation, and those with scores >39 predicted to have high risk (see Discussion).

^2^Species for which we were unable to answer >5 questions had insufficient data available to develop a score or risk status.
