# Supplementary material for: Weed Risk Assessment for Aquatic Plants: Modification of a New Zealand System for the United States
Source: PLoS One. 2012 Jul 13;7(7):e40031. doi: 10.1371/journal.pone.0040031 (PMC3396638; doi:10.1371/journal.pone.0040031)
Supplement: Table S5 — USAqWRA system results for the 20 validation species. (DOC) [file pone.0040031.s005.doc]

**Table S5.** USAqWRA system results for the 20 validation species.

| **Species name** | **U.S. *a priori* classification** | **U.S. Score** | **U.S. Risk^1^ using threshold score = 31** | **U.S. Risk^1^ using 31-39 score range for further evaluation** |
| --- | --- | --- | --- | --- |
| *Azolla pinnata* subsp. *asiatica* | Invader | 65 | High | High |
| *Glossostigma cleistanthum* | Invader | 52^2^ | High | High |
| *Glyceria declinata* | Invader | 59^2^ | High | High |
| *Ludwigia grandiflora* subsp. *hexapetala* | Invader | 74 | High | High |
| *Luziola subintegra* | Invader | 37^2^ | High | Evaluate Further |
| *Marsilea minuta* | Invader | 50 | High | High |
| *Marsilea mutica* | Invader | 42 | High | High |
| *Nymphoides cristata* | Invader | 43^2^ | High | High |
| *Rorippa amphibia* | Invader | 35 | High | Evaluate Further |
| *Salvinia molesta* | Invader | 72 | High | High |
| *Blyxa japonica* | Non-invader | 24 | Low | Low |
| *Cabomba aquatica* | Non-invader | 31 | High | Evaluate Further |
| *Elatine alsinastrum* | Non-invader | 18 | Low | Low |
| *Eleocharis multicaulis* | Non-invader | 21 | Low | Low |
| *Groenlandia densa* | Non-invader | 17 | Low | Low |
| *Isoëtes velata* | Non-invader | 18 | Low | Low |
| *Luronium natans* | Non-invader | 28 | Low | Low |
| *Myriophyllum oguraense* | Non-invader | 16 | Low | Low |
| *Nymphaea rubra* | Non-invader | 15 | Low | Low |
| *Potamogeton lucens* | Non-invader | 17 | Low | Low |

**^1^** Risk is assessed using both a single threshold score of 31 and higher identifying species with high risk of becoming invasive, and a dual threshold such that species with scores < 31 predicted to have low risk of becoming invasive, those with scores between 31 and 39 require further evaluation, and those with scores >39 predicted to have high risk (see Discussion).

^2^Some impact data from U.S. populations were used for assessment.
